# Low-Molecular Weight Heparin Increases Circulating sFlt-1 Levels and Enhances Urinary Elimination

**DOI:** 10.1371/journal.pone.0085258

**Published:** 2014-01-21

**Authors:** Henning Hagmann, Verena Bossung, Abdel Ali Belaidi, Alexander Fridman, S. Ananth Karumanchi, Ravi Thadhani, Bernhard Schermer, Peter Mallmann, Guenter Schwarz, Thomas Benzing, Paul T. Brinkkoetter

**Affiliations:** 1 Division of Nephrology, Department of Internal Medicine and Center for Molecular Medicine, University of Cologne, Cologne, Germany; 2 Department of Obstetrics and Gynecology, University of Cologne, Cologne, Germany; 3 Department of Biochemistry, University of Cologne, Cologne, Germany; 4 Howard Hughes Medical Institute and Department of Medicine, Beth Israel Deaconess Medical Center and Harvard Medical School, Boston, Massachusetts, United States of America; 5 Division of Nephrology, Department of Medicine, Massachusetts General Hospital, Harvard Medical School, Boston, Massachusetts, United States of America; Medical Faculty, Otto-von-Guericke University Magdeburg, Medical Faculty, Germany

## Abstract

**Rationale:**

Preeclampsia is a devastating medical complication of pregnancy which leads to maternal and fetal morbidity and mortality. While the etiology of preeclampsia is unclear, human and animal studies suggest that excessive circulating levels of soluble fms-like tyrosine-kinase-1 (sFlt-1), an alternatively spliced variant of VEGF-receptor1, contribute to the signs and symptoms of preeclampsia. Since sFlt-1 binds to heparin and heparan sulfate proteoglycans, we hypothesized that the anticoagulant heparin, which is often used in pregnancy, may interfere with the levels, distribution and elimination of sFlt-1 *in vivo*.

**Objective:**

We systematically determined serum and urine levels of angiogenic factors in preeclamptic women before and after administration of low molecular weight heparin and further characterized the interaction with heparin in biochemical studies.

**Methods and Results:**

Serum and urine samples were used to measure sFlt-1 levels before and after heparin administration. Serum levels of sFlt-1 increased by 25% after heparin administration in pregnant women. The magnitude of the increase in circulating sFlt-1 correlated with initial sFlt-1 serum levels. Urinary sFlt-1 levels were also elevated following heparin administration and levels of elimination were dependent on the underlying integrity of the glomerular filtration barrier. Biochemical binding studies employing cation exchange chromatography revealed that heparin bound sFlt-1 had decreased affinity to negatively charged surfaces when compared to sFlt-1 alone.

**Conclusion:**

Low molecular weight heparin administration increased circulating sFlt1 levels and enhanced renal elimination. We provide evidence that both effects may be due to heparin binding to sFlt1 and masking the positive charges on sFlt1 protein.

## Introduction

In preeclampsia, generalized endothelial dysfunction leads to a severe disorder resulting in proteinuria, hypertension, progressive edema, headaches, seizures and severe neurologic and hepatic complications [1]. Proteinuria and hypertension after the 20^th^ week of gestation are the clinical hallmarks of the condition and used for making the clinical diagnosis of preeclampsia. Recent studies suggest, that circulating anti-angiogenic substances such as a soluble splice variant of the VEGF-receptor Flt-1 (sFlt-1) and a soluble form of the TGF-β-receptor Endoglin, soluble endoglin (sEng) act in concert to induce endothelial dysfunction and the maternal syndrome of preeclampsia [2]–[4]. Importantly, serum levels of these anti-angiogenic substances precede the clinical signs of preeclampsia by 5–6 weeks [4], [5] suggesting that targeting these pathways may be useful for treating or preventing this disorder.

During the last five years, several studies have used commercially available ELISA assays to evaluate the plasma or serum levels of pro- and anti-angiogenic factors for both diagnosis and prediction of preeclampsia [6]. In particular, determination of sFlt-1 and its binding partner PlGF have proven reliable for prediction and early diagnosis of preeclampsia [4], [7]–[12]. Automated assays to measure sFlt-1 and PlGF are now available and allow for expeditious routine screening of preeclampsia in an in-patient and out-patient setting [12]–[14].

However, very little is known about sFlt-1 kinetics and potential serum or plasma interfering factors and medical conditions that may influence sFlt-1 levels and elimination. Recent reports indicated that acute administration of high doses of heparin may lead to increased plasma levels of sFlt-1 up to 50-fold [15], [16]. This is of particular interest as the administration of heparin is often used in patients with thrombophilia, who are at risk for preeclampsia and also hospitalized pregnant patients to prevent DVTs (deep venous thrombosis) [17]–[23]. In contrast to other reports, Rosenberg and colleagues reported that heparin did not affect sFlt1 mRNA expression, or synthesis from trophoblast tissue [24], [25]. In their studies, Rosenberg et al. did not specifically investigate shedding or proteolytic cleavage. However, other groups have suggested that the increased levels of circulating sFlt-1 are likely due to heparin-mediated mobilization of sFlt-1 from heparin sulfate proteoglycans present on cell surface or in the extracellular matrix [15], [16].

We performed serial measurements of sFlt-1 and PlGF serum levels in 11 patients with suspected preeclampsia who received daily weight adjusted low molecular weight heparin (LMWH) for DVT-prophylaxis to investigate the influence of heparin administration on the diagnostic assays of sFlt-1 and PlGF. In addition we analyzed urinary excretion of sFlt-1 in the presence and absence of heparin to learn about potential routes and modalities of sFlt-1-elimination.

## Methods

### Patient population

We enrolled 11 patients between 18 and 45 years of age between April 2011 and June 2012 who were admitted to the University Hospital Cologne with suspected or clinically evident preeclampsia and who received subcutaneous low molecular weight heparin (0,5 mg enoxaprin per kg bodyweight daily) for DVT prophylaxis. Protocols were reviewed and approved by the ethics committee of the University Hospital Cologne (protocol number 10–238). Written informed consent was obtained from all participants. Gestational age was estimated by menstrual history and ultrasonography. Preeclampsia was defined as new onset of hypertension >140/90 and proteinuria >0.3 g protein/g creatinine in spot urine samples or total protein excretion >300 mg protein/24 hrs. HELLP syndrome (hemolysis elevated liver enzymes, low platelets) was defined according to established criteria [26].

### Blood and urine sampling

Blood and urine samples were drawn 2 hrs before administration of enoxaparin-sodium, at the time of subcutaneous enoxaparin injection and 2 hrs, as well as 12 hrs after LMWH (Figure S1). Blood samples were obtained by venipuncture from the cubital vein using serum separation tubes. After 10 min centrifugation at 1.200 rcf in an Eppendorf 5810R swinging bucket centrifuge samples were analysed within 2 hrs after the blood draws, and remaining sample was stored at −80°C. Urine samples were obtained by the clean catch method as spot urine samples. Samples were spun at 600 rcf for 10 min, the supernatant was analysed directly, and remaining sample was stored at −80°C. Other parameters were assessed as per standard protocols of the University Hospital Cologne.

### sFlt-1 and PlGF measurements

Serum levels of sFlt-1 and PlGF were determined by using the Elecsys® assays (Roche) as previously described [12]. To determine urinary sFlt-1, we employed the Quantikine® human sFlt-1 ELISA (R&D) as described elsewhere [27]. For *in vitro* experiments enoxaprin was spiked into pre-treatment serum samples at a final concentration of 10 mg/l. The samples were then analyzed with the Elecsys® assays.

### Velocity gradient centrifugation

A 2 ml discontinuous sucrose gradient (40–5%) was layered on top of a 60% sucrose cushion in an ultracentrifuge tube (Beckman, Fullerton, CA). Serum was diluted to equal parts with TBS buffer, added on top of the gradient, and subjected to centrifugation for 22 h at 215,000×***g*** at 4°C in a Beckman SW-60Ti rotor. After centrifugation 22 fractions (200 µl each) were collected, and analyzed by SDS/PAGE. Flt-1 specific monoclonal antibody (R&D) was used for immunoblot.

### Affinity purification of serum samples

Serum purification consisted of a cation exchange chromatography step and was performed using the Mono S HR 5/5 column (Amersham Bioscience). Gradient separation was performed on an FPLC system (Äkta, GE Healthcare) using a buffer A (50 mmol/l Tris, pH 7.3) and a buffer B (50 mmol/l Tris, pH 7.3, 1 mol/l NaCl). Serum samples were diluted 1∶1 with buffer A and loaded in a total volume of 1 ml on the pre-equilibrated column (Flow rate: 2 ml/min, 11% B). Serum fractions were separated using an increasing gradient of buffer B (11 to 100% over 30 ml). Fractions were collected during all purification steps and stored at −20°C upon analysis. All chromatography steps were performed at 4°C and all different serum samples were analyzed using the same buffers and an automated chromatography method allowing identical separation conditions.

### Statistical analysis

Continuous variables were analyzed by Student's ***t***-test and regression analysis was performed using logistic regression techniques using GraphPad Prism version 4.00 (GraphPad Software, San Diego, CA). All ***P*** values were two-tailed, and a ***P*** value<0.05 was considered statistically significant.

## Results

We studied 11 patients admitted with suspected or established preeclampsia between 27 to 38 weeks of gestation, who were receiving low molecular weight heparin. Patient characteristics and laboratory values upon admission are outlined in **Table 1**. In the study blood and urine samples were drawn 2 hrs before administration of LMWH (enoxaparin-sodium), at the time of subcutaneous LMWH injection and 2 hrs, as well as 12 hrs after LMWH (Figure S1). The heparin dose was adapted to patient weight ranging from 40 mg to 100 mg enoxaparin subcutaneously according to standard recommendations.

**Table 1:** Patient characteristics on admission.

Characteristics	Pat_1	Pat_2	Pat_3	Pat_4	Pat_5	Pat_6	Pat_7	Pat_8	Pat_9	Pat_10	Pat_11
Maternal Age (yrs)	35	29	34	34	29	28	24	38	44	33	27
Gestational Age (weeks+days)	32+6	27+1	38+3	33+4	35+5	35+2	32+0	35+1	37+4	30+4	31+1
Systolic Blood Pressure (mmHg)	145	149	142	151	142	160	156	148	165	162	148
Diastolic Blood Pressure (mmHg)	97	102	95	92	92	102	101	97	101	92	101
Urine protein/creatinine ratio (mg/g)	234	<0.04	741	632	241	1758	606	1889	196	338	15812
sFlt-1 plasma level (pg/ml)	8932	6501	3179	6427	2790	5339	24250	20514	6319	6596	8279
PlGF plasma level (pg/ml)	29.8	31.6	155.8	306.1	184.8	156.7	134.7	129.0	76.3	83.3	48,6
Hemoglobin (g/L)	11.5	13.0	10.7	10.1	11.0	11.0	11.4	12.6	13.3	13,4	12.1
Platelets (×10^9^/L)	69	209	203	286	184	185	150	148	237	300	263
Creatinine (µmol/L)	0.77	0.56	1.02	0.67	0.49	0.66	0.90	0.99	0.66	0.80	0.64
SGOT (U/L)	128	24	17	17	27	22	175	24	19	15	20
SGPT (U/L)	107	21	11	11	24	19	131	17	11	14	13
LDH (U/L)	327	182	203	179	177	268	260	223	184	178	226
Diagnosis	HELLP	IUGR, PIH	DM1+late PE	twins, PE	PIH	PE	HELLP	twins, PE	PIH	PE	PE

Physical parameters and laboratory values were assessed upon admission in all patients. DM1 = diabetes mellitus type 1; IUGR = intra uterine growth restriction; PE = preeclampsia; PIH = pregnancy induced hypertension.

### Serum sFlt-1 levels increase after heparin administration

**Figure 1:**
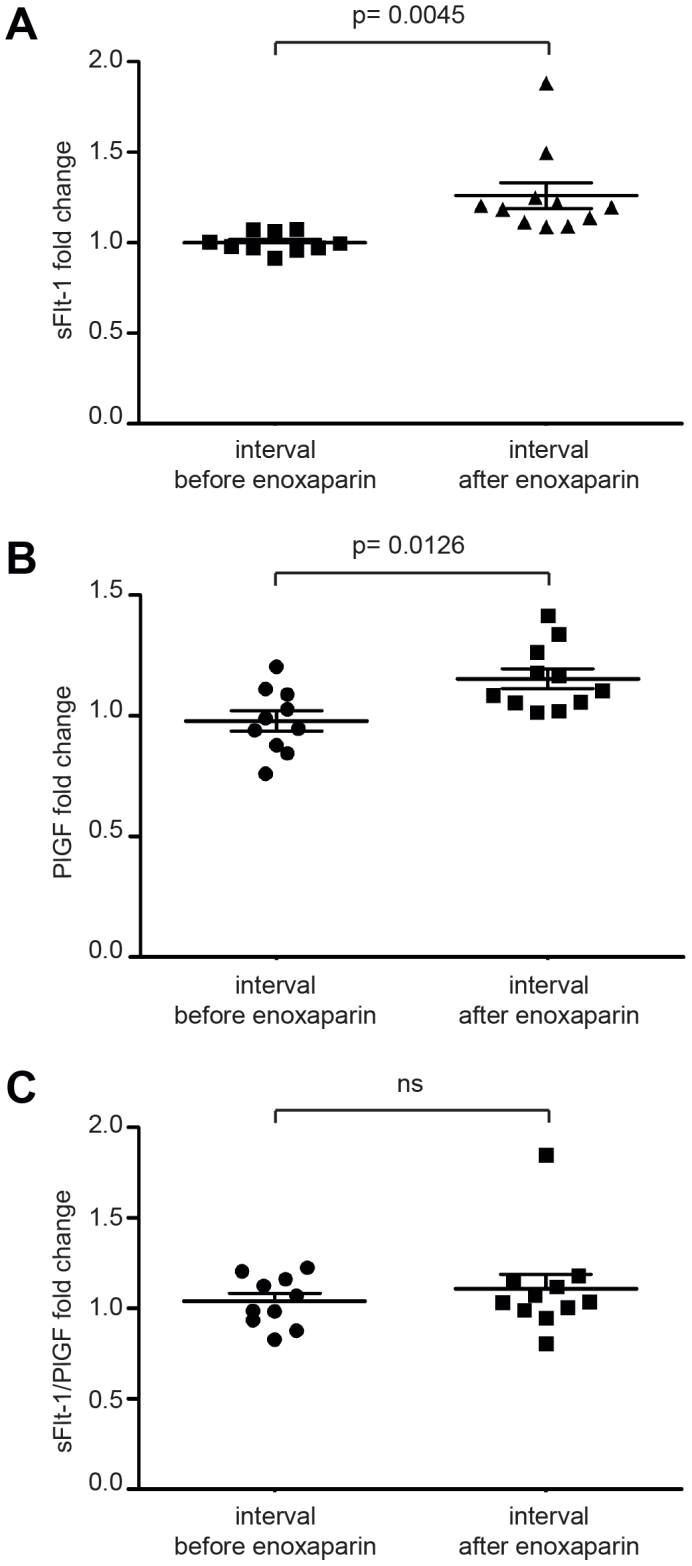
sFlt-1 and PlGF serum levels increase after the administration of low molecular weight heparin. 2-1 levels increase to 1.26 fold on average (95% CI (interval before LMWH) 0,9627–1,040; 95% CI (interval after LMWH) 1.103–1.418; p = 0.0045) (**A**), PlGF levels increase to 1.15 fold on average (p = 0.0126) (**B**), the resulting increase of sFlt-1/PlGF ratio does not reach statistical significance (**C**).

sFlt-1 serum levels at baseline and at the time of heparin administration were within the detectable range (median 6501 pg/ml; 5829 pg/ml–8605.5 pg/ml, 25^th^–75^th^ centile) and positively correlated with the clinical diagnosis of preterm preeclampsia and HELLP syndrome [5], [8], [9]. To assess alterations of sFlt-1 and PlGF serum levels following LMWH administration we determined relative changes in the interval “before enoxaparin”, i.e. sFlt-1 and PlGF serum values 2 hrs before treatment, at the time of treatment, and 2 hrs after enoxaparin treatment. sFlt-1 serum levels 2 hrs before and at the time of treatment revealed no significant changes. Hence the difference between the first two readings in any individual patient may serve as a valid internal control. 2 hrs after the administration of LMWH sFlt-1 serum levels increased by 26.0% on average (mean 1.26; p = 0.0045) (Fig. 1A). PlGF serum levels showed a more moderate increase of 15.4% (mean 1.154; p = 0.0126) (Fig. 1B). Alterations in the sFlt-1/PlGF ratio were consequently small and did not reach statistical significance (mean 1,119; p = 0.3031, Fig. 1C). Changes of sFlt-1, PlGF and sFlt-1/PlGF for individual patients in the study are shown in Figure S2A–C. 12 hours after administration of LMWH serum sFlt-1 declined to almost the baseline levels (fold change compared to baseline: mean 1.096; p = 0.4594). To control for effective application of LMWH we determined blood levels of anti-activated Factor X activity. As expected, anti-Xa levels retraced the amount of circulating LMWH (Figure S3) whereas no significant difference was found in partial thromboplastin time (mean fold change 1.08, p = 0.0720).

### Determination of PlGF is affected by low molecular weight heparin *in vitro*

**Figure 2:**
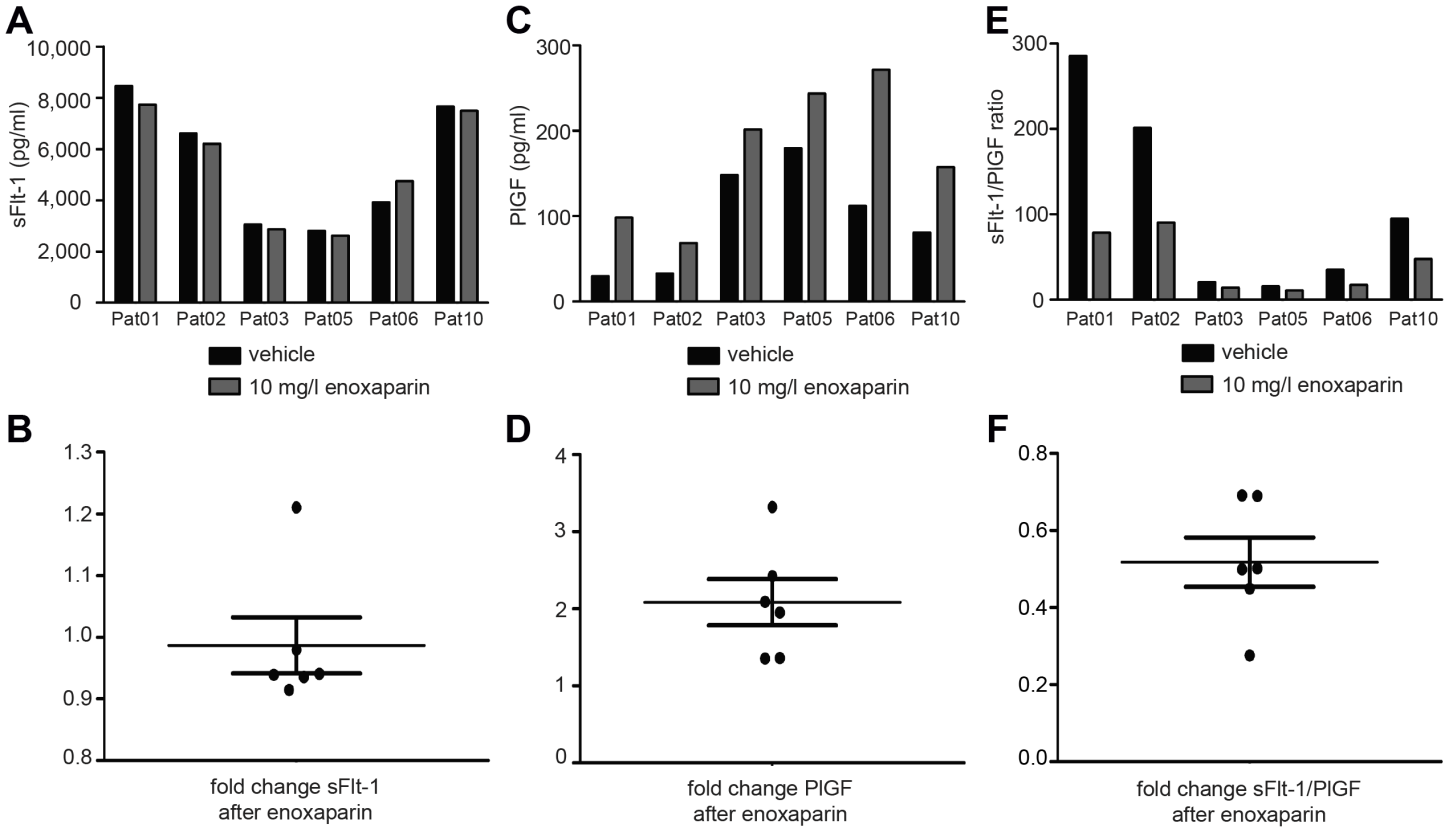
Addition of low molecular weight heparin *in vitro* affects PlGF measurements in the Elecsys Assay. Assessment of serum levels after the administration of enoxaparin or vehicle *in vitro* shows only marginal differences for sFlt-1 (**A+B**). In contrast PlGF levels appear falsely high after enoxaparin (**C+D**). Consequently the sFlt-1/PlGF ratio appears decreased (**E+F**).

To control for assay artefacts, we added LMWH to pre-treatment serum samples at a final concentration of 10 mg/l *in vitro* and determined sFlt-1 and PlGF in the Elecsys® assay. The heparin concentration of 10 mg/l used in the *in vitro* studies was the result of a careful estimation of plasma levels. The volume of distribution of heparin equals the plasma volume which contributes to approximately 5% of the total bodyweight. A person of 70 kg body weight will have approximately 3.5 liters of plasma. The same patient would receive 35 mg enoxaparin (weight-adapted dose). The resulting plasma concentration of enoxaparin in this setting is approximately 10 mg/l. While sFlt-1 levels were not significantly altered following LMWH application (mean 0.9865; SEM 0.046) (Fig. 2A), PlGF levels were significantly increased (mean 2.084; SEM 0.309) (Fig. 2B). Consequently, the sFlt-1/PlGF ratio appeared decreased. These findings ruled out assay artefacts for the determination of sFlt-1 levels; however heparin interfered with the PlGF assay. We therefore chose to further pursue the *in vivo* effects of heparin on sFlt-1 levels.

### The magnitude of sFlt-1 increase correlates positively with initial sFlt-1 serum levels

**Figure 3:**
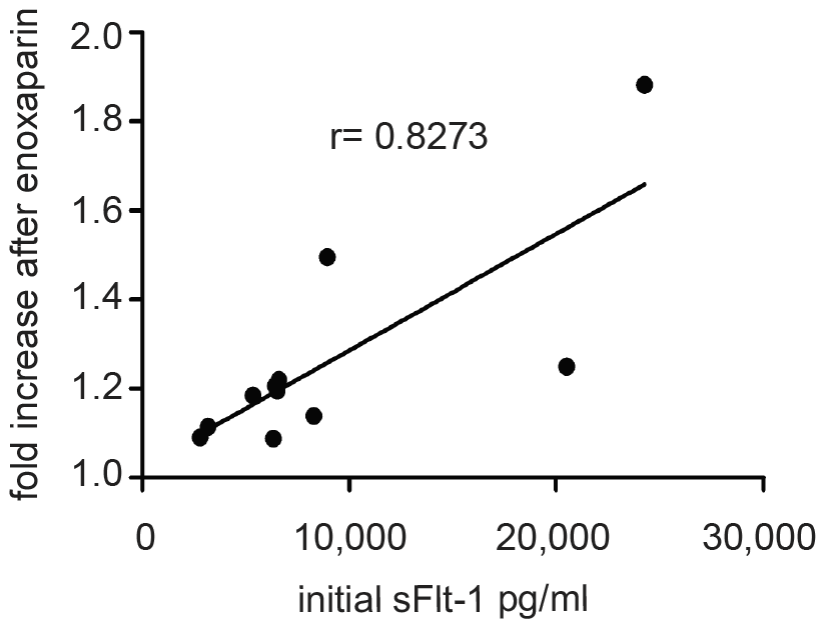
The rate of increase in serum sFlt-1 shows positive correlation with the initial serum sFlt-1 levels. Regression analysis of the alteration in serum sFlt-1 levels following enoxaparin (fold change) and initial sFlt-1 serum levels yields positive correlation with the Spearman correlation coefficient r = 0.8273 (95%CI = 0.4348 to 0.9556; p = 0.0014).

The study population was quite diverse with regard to disease severity and sFlt-1 levels. Some of the patients enrolled showed low sFlt-1 serum levels of 3,000–4,000 pg/ml which were considered borderline normal levels at their gestational age, while other patients enrolled suffered from severe preeclampsia with sFlt-1 serum levels above 20,000 pg/ml. Interestingly, the magnitude of sFlt-1 increase following LMWH administration seemed to depend on the initial sFlt-1 serum level (Figure S2A). To determine a potential correlation between baseline sFlt-1 levels and the amount of increase after heparin we performed regression analysis using the Spearman coefficient. The rate of increase correlated with initial sFlt-1 levels with a Spearman correlation coefficient of 0.8273 (95%CI = 0.4348 to 0.9556; p = 0.0014) (Fig. 3).

### Urinary sFlt-1 levels increase after heparin administration

sFlt-1 is detectable in the urine of preeclamptic patients [27]. Yet, the mechanism of renal sFlt-1 excretion has not been explored. To test whether heparin affects the elimination of sFlt-1 from the circulation, we assessed renal excretion of sFlt-1 in response to heparin treatment. Urine samples from before and after LMWH administration were analysed using a sFlt-1 specific ELISA. Patient #3 was lacking a timed urine sample two hours after treatment and therefore excluded from this analysis.

**Figure 4:**
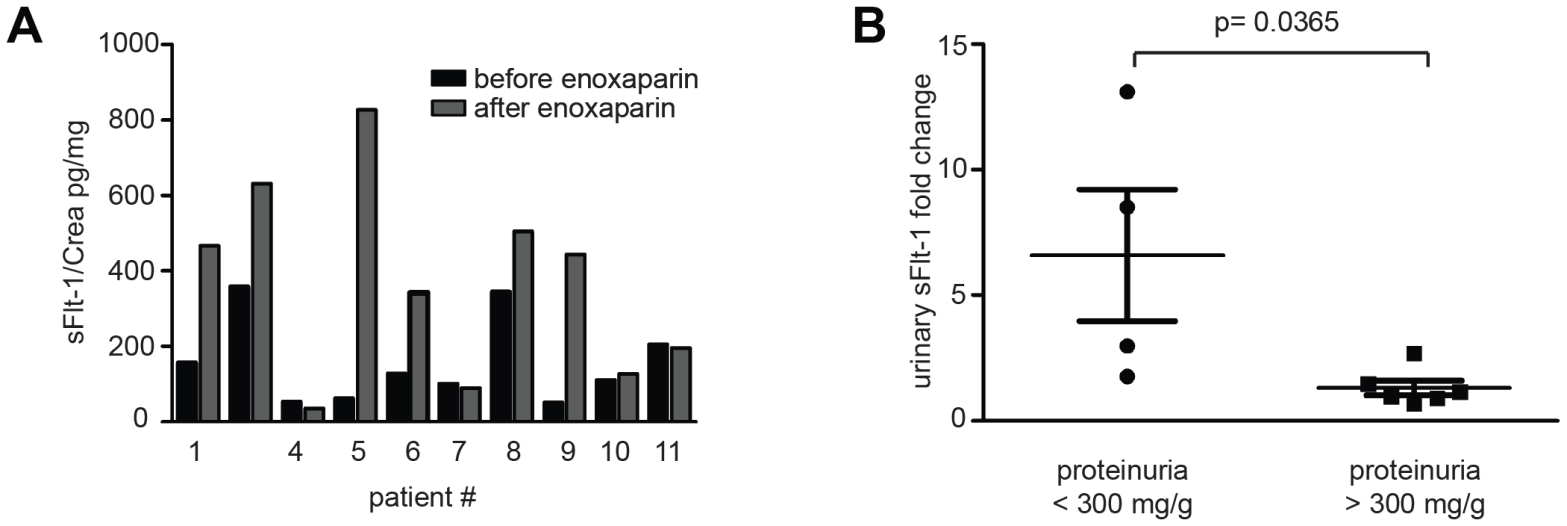
Administration of low molecular weight heparin results in elevated urinary sFlt-1 levels. Urinary sFlt-1 levels (pg/mg Crea) are increased 2 hours after administration of enoxaparin in 7 out of 10 patients (**A**). Subgroup analysis according to proteinuria at presentation (cut-off of 300 mg protein/g creatinine) reveals that patients with a proteinuria <300 mg/g respond with a significantly higher increase of urinary sFlt-1 after heparin treatment (p = 0.0365) (**B**).

Baseline levels of urinary sFtl-1 did not correlate with the extent of proteinuria in individual patients (data not shown). After heparin administration urinary levels of sFlt-1 increased in 7 out of 10 patients (Fig. 4A) compared to initial urinary sFlt-1 levels. Since sFlt-1 is a positively charged protein with isoelectric point of approximately 9.5 [28] and glomerular filtration largely depends on the intact charge selectivity of the glomerular filter, we asked whether urinary excretion of sFlt-1 in response to heparin is dependent on the integrity of the glomerular filter. We then performed subgroup analysis dividing the study population into two groups according to the grade of proteinuria using 300 mg protein/g creatinine as a cut-off. Patients with relatively lower proteinuria (Pat. #1, 2, 5, 9) and presumably preserved glomerular filter showed a significant increase of urinary sFlt-1 excretion following heparin administration (mean 6.59; SEM 2.63). In striking contrast, the subgroup with a proteinuria >300 mg/g only experienced a small increase in urinary sFlt-1 after heparin (mean 1.30; SEM 0.29) (Fig. 4B). No correlation between urinary sFlt-1excretion and initial sFlt-1 serum levels was found (Figure S4B). Addition of LMWH to serum and urine samples did not affect measurements in the R&D assay (Figure S5).

### Heparin alters charge-dependent binding properties of sFlt-1 without affecting complex size

**Figure 5:**
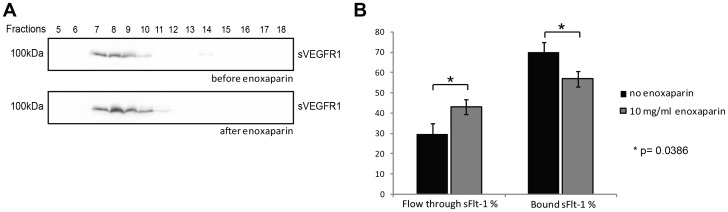
Heparin does not affect sFlt-1 protein complex size but interferes with sFlt-1 binding to negatively charged surfaces. Serum samples before and after low molecular weight heparin treatment were subjected to velocity gradient centrifugation. Western blotting of fractions 5–18 was performed using Flt-1 specific antibody (**A**). Serum samples before and after addition of 10 mg/l enoxaparin were subjected to cation exchange chromatography. In non-treated serum samples 70.14% of sFlt-1 binds to the column, whereas the remaining 29. 86% are found in the flow through. After enoxaparin treatment only 56.88% of sFlt-1 is bound and 43.11% appear in the flow through (p = 0.0386) (**B**).

Knowing that renal elimination of sFlt-1 can be increased after heparin therapy in patients with intact glomerular barrier, we set out to further characterize the molecular basis of this effect in biochemical studies. We tested the hypothesis that due to their opposite charges, heparin liberates sFlt-1 from larger multi-molecular complexes by charge dependent interaction. Monomeric sFlt-1 could then potentially be filtered via glomerular barrier or be secreted into the proximal tubule. In order to experimentally substantiate changes in complex size we employed velocity gradient ultra-centrifugation on serum samples to separate native complexes of serum proteins according to their density before and after administration of heparin (Fig. 5A). After separation of up to 22 fractions on SDS-PAGE and immunoblot analysis using anti-Flt-1 specific antibody, sFlt-1 was detected in the fractions 4–17 in samples of both before and after treatment. This led us to infer that sFlt-1 complex size is not affected by heparin administration.

In addition to size selectivity, selective exclusion of proteins from the glomerular filtration is highly dependent on the molecular charge [29]–[33]. As sFlt-1 is highly positively charged at physiologic pH, it is likely to bind to the negative charge of the glomerular basement membrane. LMWH may mask the positive charge in the sFlt-1 molecule and, may facilitate glomerular passage. To analyse changes in complex charge of sFlt-1 we subjected serum samples before and after addition of 10 mg/l enoxaparin to cation exchange chromatography. To compare binding properties of sFlt-1 before and after the administration of heparin we allowed binding of sFlt-1 to the resin at a concentration of 110 mmol/L NaCl. Following elution with increasing salt concentrations sFlt-1 was quantified in the flow through and in the bound fractions using the Quantikine ELISA. In untreated serum samples on average 70.14% of sFlt-1 bound to the cation exchange matrix. After addition of enoxaparin at a concentration 10 mg/l mimicking the *in vivo* situation after LMWH treatment only 56.88% of the total sFlt-1 in the sample bound to the column. These results were well complemented by analysis of the sFlt-1 concentration in the flow through (Fig. 5B).

## Discussion

Measurements of circulating angiogenic factors such as sFlt-1 and PlGF are rapidly evolving as tests for aid in diagnosis of preeclampsia [6]. There is a large body of evidence that these biomarkers may help not only in the early diagnosis of preeclampsia, but also help with prediction of adverse maternal and fetal outcomes [4], [5], [9], [34]–[39]. Yet much has to be learned with regard to influencing variables and characteristics of different testing platforms. In addition, understanding the biochemical properties and kinetics of sFlt-1 may allow us to identify novel therapeutic targets for preeclampsia.

In this study, we sequentially measured blood and urine sFlt-1 levels before and after administration of LMWH for DVT-prophylaxis in preeclamptic patients. Blood samples drawn 2 hrs prior and at the time of heparin injection in each patient served as control. We show that circulating sFlt-1 levels are increased following administration of LMWH. Our findings not only support previous reports by other groups in [25] but also reveal a positive correlation between the initial sFlt-1 levels and the sFlt-1 increase following LMWH. As has been reported previously, we also observed that sFlt-1 concentrations declined to the baseline value within 12 hrs after administration of LMWH suggesting that these effects are temporally related to heparin therapy [15]. To exclude the possibility that the changes in sFlt-1 levels may be due to assay interference with heparin, we spiked low molecular weight heparin to serum samples *ex vivo* and found no interference. However, PlGF levels were elevated two fold following *ex vivo* heparin treatment suggesting, that PlGF levels may not be reliably used in patients receiving heparin therapy due to interference with the assay. Taken together, we conclude that the effect of heparin on sFlt-1 serum levels is highly dependent on the initial sFlt-1 levels and the timing of heparin use. Our data has important implication for using sFlt1 and PlGF assays in patients receiving heparin and would advise against testing any sFlt-1 and PlGF levels until heparin has completely cleared from the blood circulation.

We speculate that upon heparin treatment sFlt-1 bound to heparan sulfate proteoglycans of the extracellular matrix is mobilized into the circulation, as suggested by others [16]. Patients with high sFlt-1 levels at baseline bear a higher overall sFlt-1 load, i.e. besides higher serum levels also the extracellular matrix is highly saturated with sFlt-1 molecules. Administration of exogenous heparin with its almost exclusive intravascular distribution and its high negative charge adds additional sFlt-1 binding sites to the intravascular compartment. More sFlt-1 bound to extracellular matrix may translocate into the blood stream binding to circulating heparin molecules which is detected on the diagnostic assays. This is consistent with the observation of displacement of sFlt-1 from pericellular glycosaminoglycans in aortic arch - and umbilical vein explants as well as from human placental villi in culture [15], [16]. Binding of sFlt-1 to heparin is most likely mediated by positively charged stretches of basophilic aminoacids in the third and fourth immunoglobulin-like domain of sFlt-1 [28], [40].

Due to its high molecular weight and positive charge, sFlt-1 is expected to be excluded from glomerular filtration. sFlt-1 has been shown to accumulate in close proximity to the glomerular endothelium with the negatively charged constituents of the glomerular basement membrane where it may interfere with the VEGF-dependent cross-talk between podocytes and endothelial cells. This may very well explain the early and pronounced renal phenotype of the multi-systemic disease of preeclampsia. Nevertheless, there are reports that sFlt-1 can be detected in the urine during active preeclampsia and may serve as diagnostic marker [27], [41]. In our study, we could confirm the previously published range of sFlt-1 levels in urine samples of patients with preeclampsia and HELLP syndrome. However, we did not find any correlation between serum and urine levels of sFlt-1. Surprisingly, we found a heterogenous response of urinary sFlt-1 levels to heparin treatment. Subgroup analysis revealed that patients with lower or absent proteinuria and presumably intact glomerular filter responded with more dramatic urinary excretion of sFlt-1 following heparin administration. In patients with significant glomerular damage and proteinuria this effect is lost. In the latter patients, serum sFlt-1 is detected at much higher levels. Although we cannot rule out *de novo* synthesis of sFlt-1 in tubular or glomerular cells or tubular secretion, this data strongly suggests that urinary excretion of sFlt-1 is dependent on molecular charge. sFlt-1 filtration is enhanced after heparin in those patients with an intact glomerular filter. In patients with a compromised glomerular filtration barrier charge selectivity is lost and heparin administration will not lead to an increment of filtered sFlt-1. Interestingly, sFlt-1 excretion and the response to LMWH did not correlate with initial serum sFlt-1 levels.

To understand the underlying mechanism of increased serum levels and altered renal elimination of sFlt-1 following heparin we addressed the question whether heparin could influence size and/or molecular charge of the sFlt-1 protein complex.

Our biochemical experiments showed that heparin treatment did not affect the size of the sFlt-1 protein complex. This excluded the possibility that heparin treatment led to the disassembly of larger multi-molecular sFlt-1 complexes. Employing cation exchange chromatography on serum samples before and after addition of LMWH we could show that heparin reduces the affinity of sFlt-1 to negatively charged surfaces. This suggests that LMWH treatment *in vivo* weakens the binding of sFlt-1 to fixed negative charges of the glomerular basement membrane and thus potentially facilitates glomerular filtration of sFlt-1.

Our study has certain limitations. The sample size is small. This is a general problem in studies on preeclamptic patients recruited during in-patient setting. Once these patients require hospital admission, rapid deterioration in the medical condition or imminent delivery impedes enrolment into clinical studies. Therefore cooperation of multiple centers and out-patient clinics with similar treatment protocols is warranted when studying patients with preeclampsia. In addition our biochemical studies can only provide indirect proof of an alteration of sFlt-1 complex charge. Further biochemical and *in vivo* studies are warranted to establish that renal elimination of sFlt-1 indeed depends on protein-charge. Different modifications of recombinant sFlt-1 with alterations of charged residues should be generated to study sFlt-1 biodistribution and renal excretion e.g. in a mouse model. Employing angiogenic factors like sFlt-1 and PlGF for clinical use for the prediction and diagnosis of preeclampsia as well as validating therapeutic approaches warrants complete understanding of the characteristics and influencing variables of those factors *in vivo* and *in vitro*.

In summary, we conclude from our observations that (i) acute administration of low molecular weight heparin leads to increased sFlt-1 serum levels (ii) Heparin bound sFlt-1 may be more easily eliminated into the urine by the kidney. The source of increased circulating sFlt1 following low molecular weight heparin therapy is unknown. Whether enhanced urinary excretion of sFlt-1 following heparin therapy is a result of glomerular filtration or tubular secretion is also yet to be determined.

## Supporting Information

Figure S1 Study scheme. Blood samples (red triangles) were drawn 2 hours prior and directly before sub cutaneous injection of low molecular weight heparin (enoxaparin-sodium). In succession blood was obtained 2 hours and 12 hours after heparin administration.
(TIF)

Figure S2 Alterations of serum sFlt-1, serum PlGF and sFlt-1/PlGF ratio in individual patients. sFlt-1, PlGF and sFlt-1/PlGF was determined in the serum of the study patients 2 hours prior, directly before and 2 hours after enoxaparin treatment. The figures shows fold change of sFlt-1 (**A**), PlGF (**B**) and sFlt-1/PlGF (**C**) in the interval before and after treatment.
(TIF)

Figure S3 Anti-activated Factor X levels serve as a control for the administration of low molecular weight heparin. Anti-Xa was determined with all main blood draws.
(TIF)

Figure S4 Alterations of urinary sFlt-1 in individual patients. sFlt-1 was determined in the urine of 10 out of 11 study patients before and after enoxaparin treatment and normalized to urinary creatinine levels. (**A**) shows fold change of urinary sFlt-1/Crea. No correlation was found between urinary sFlt-1/Crea and initial sFlt-1 serum levels (**B**).
(TIF)

Figure S5 Addition of low molecular weight heparin *in vitro* does not affect sFlt-1 measurements using the R&D ELISA kit. Assessment of serum levels with the R&D ELISA after the administration of enoxaparin or vehicle *in vitro* shows no significant differences in sFlt-1 levels (**A and B**). There is also no significant difference seen in urinary sFlt1 levels after administration of enoxaparin or vehicle *in vitro* (**C and D**).
(TIF)
