# Supplementary figures and images for: Low-Molecular Weight Heparin Increases Circulating sFlt-1 Levels and Enhances Urinary Elimination
Source: PLoS One. 2014 Jan 21;9(1):e85258. doi: 10.1371/journal.pone.0085258 (PMC3897409; doi:10.1371/journal.pone.0085258)

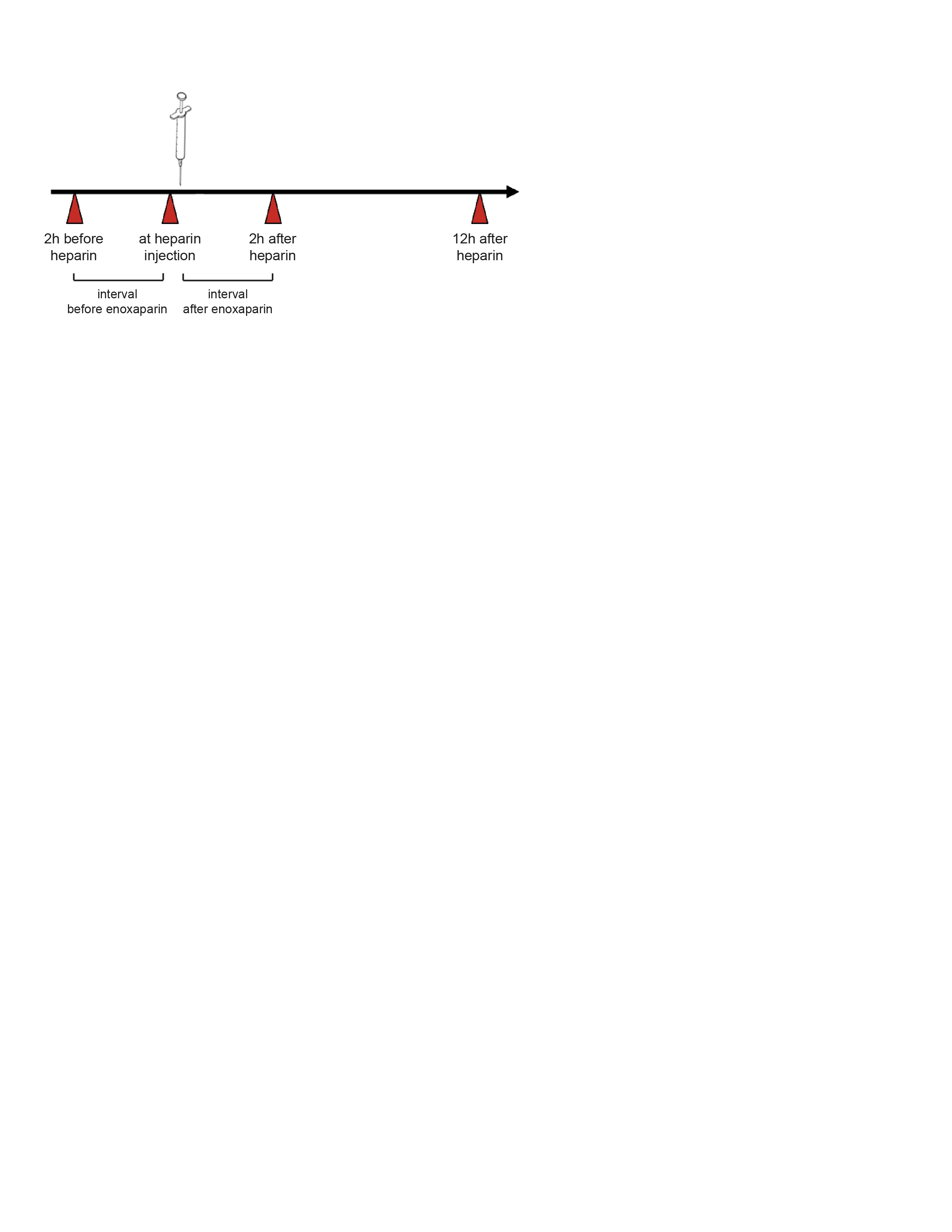

Supplement: Figure S1 — Study scheme. Blood samples (red triangles) were drawn 2 hours prior and directly before sub cutaneous injection of low molecular weight heparin (enoxaparin-sodium). In succession blood was obtained 2 hours and 12 hours after heparin administration. (TIF) [file pone.0085258.s001.tif]

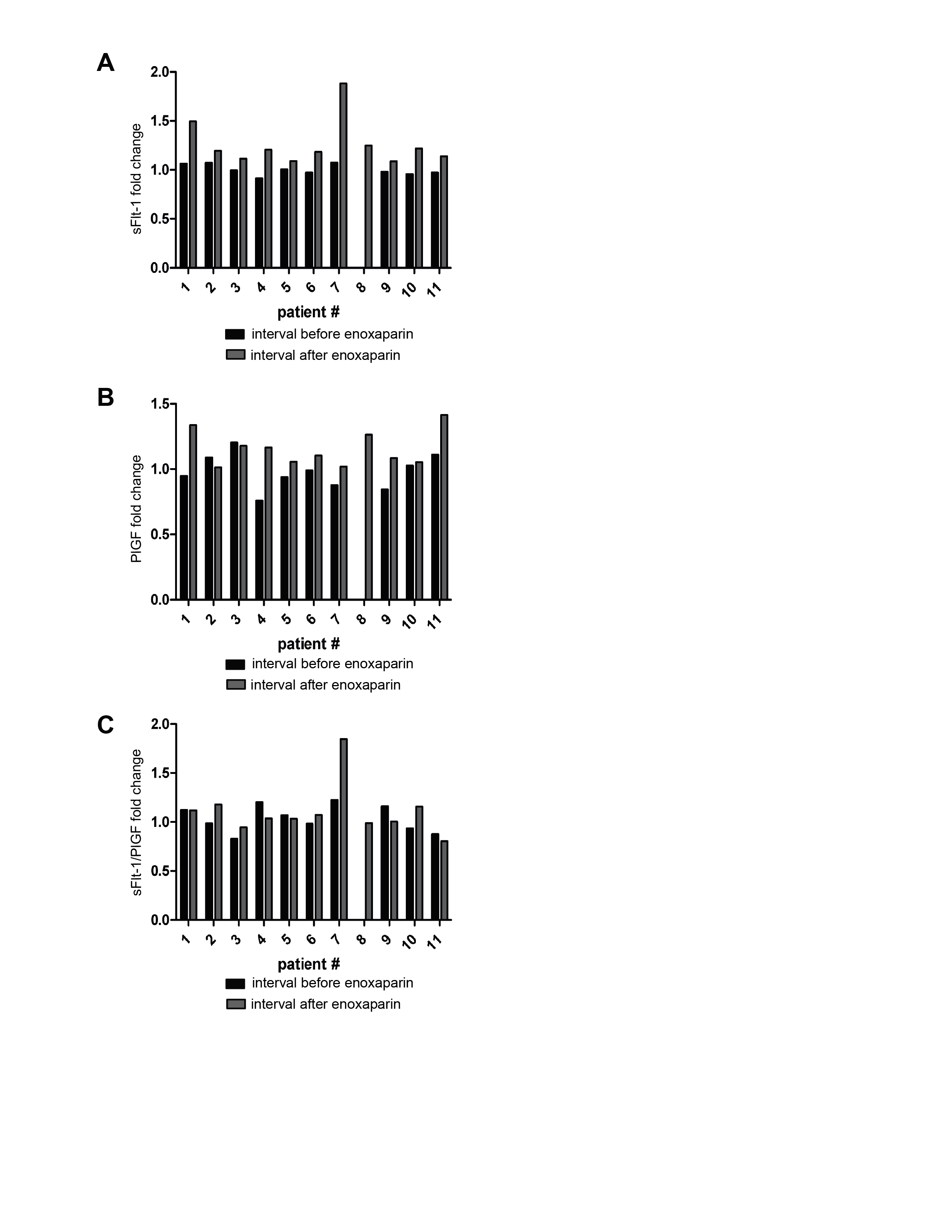

Supplement: Figure S2 — Alterations of serum sFlt-1, serum PlGF and sFlt-1/PlGF ratio in individual patients. sFlt-1, PlGF and sFlt-1/PlGF was determined in the serum of the study patients 2 hours prior, directly before and 2 hours after enoxaparin treatment. The figures shows fold change of sFlt-1 (A), PlGF (B) and sFlt-1/PlGF (C) in the interval before and after treatment. (TIF) [file pone.0085258.s002.tif]

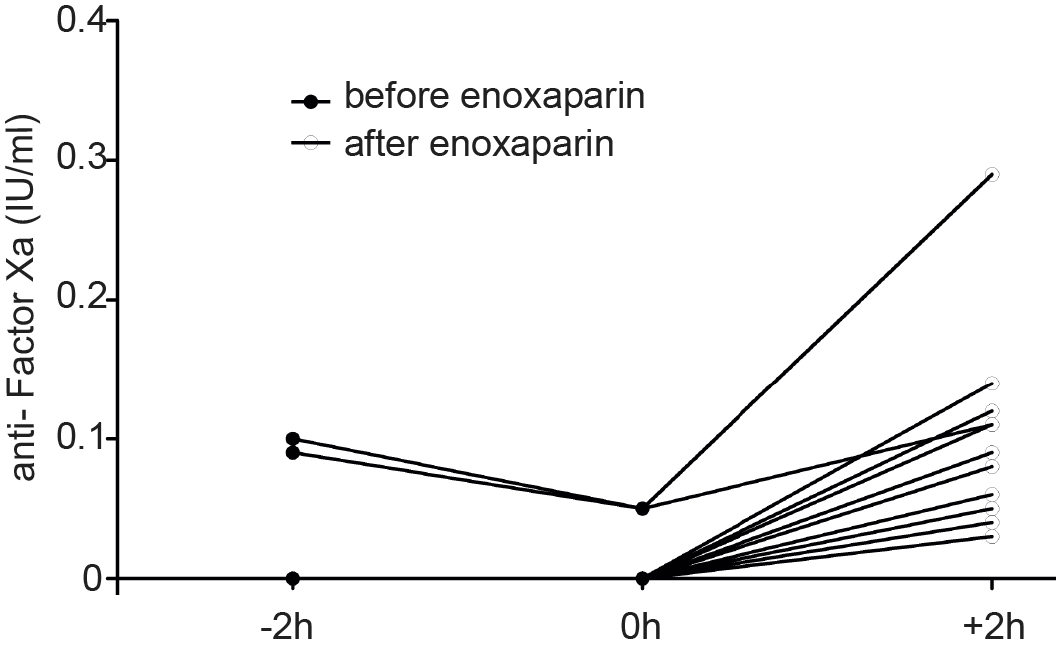

Supplement: Figure S3 — Anti-activated Factor X levels serve as a control for the administration of low molecular weight heparin. Anti-Xa was determined with all main blood draws. (TIF) [file pone.0085258.s003.tif]

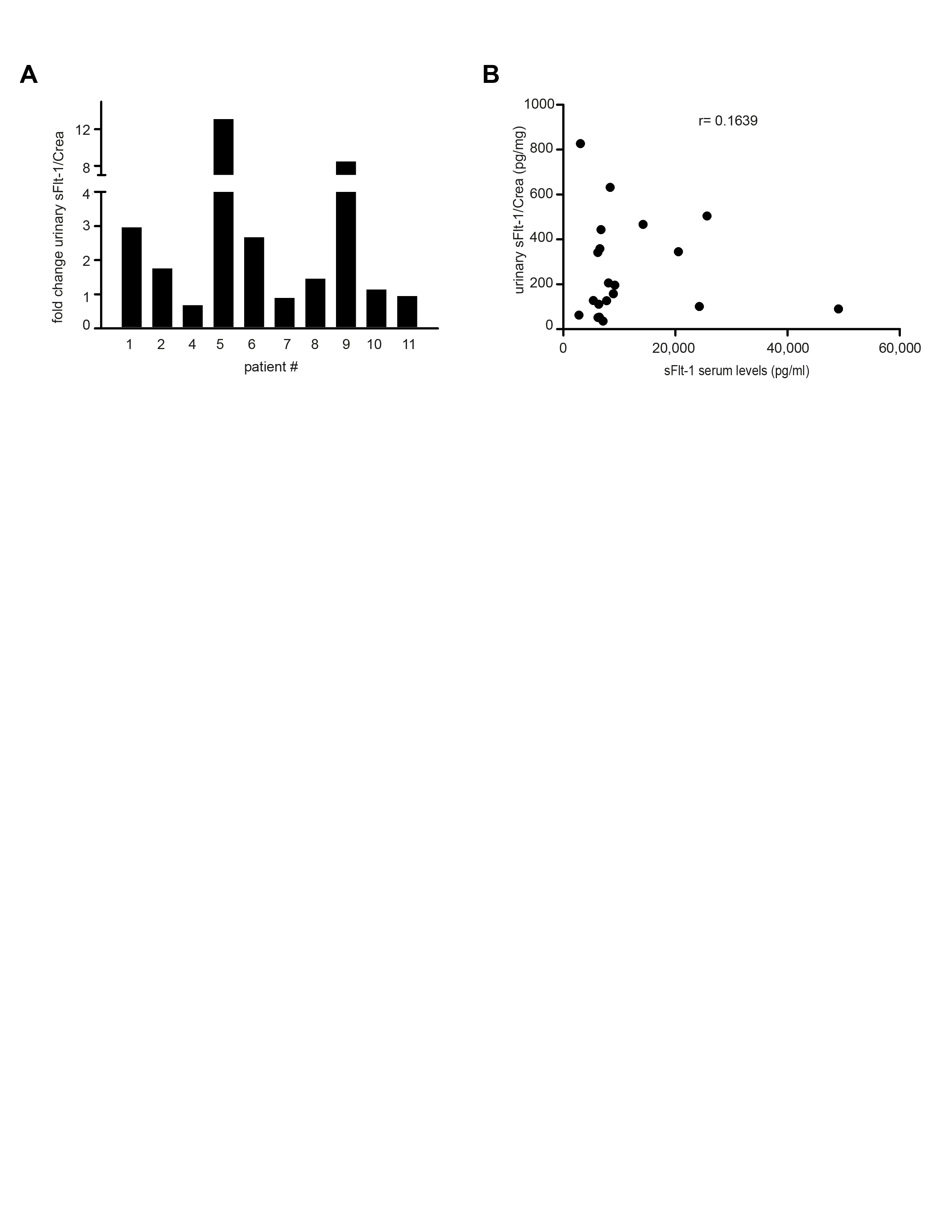

Supplement: Figure S4 — Alterations of urinary sFlt-1 in individual patients. sFlt-1 was determined in the urine of 10 out of 11 study patients before and after enoxaparin treatment and normalized to urinary creatinine levels. (A) shows fold change of urinary sFlt-1/Crea. No correlation was found between urinary sFlt-1/Crea and initial sFlt-1 serum levels (B). (TIF) [file pone.0085258.s004.tif]

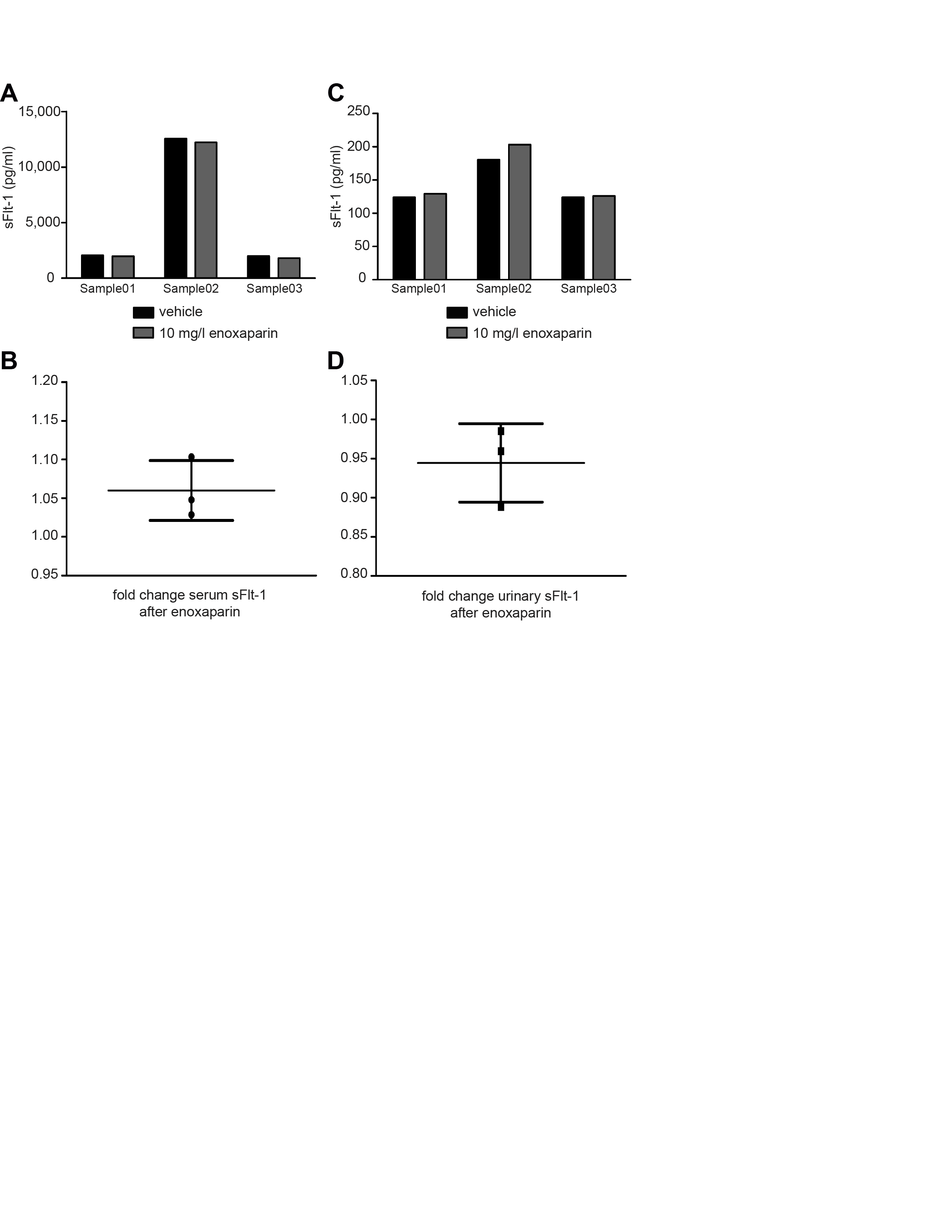

Supplement: Figure S5 — Addition of low molecular weight heparin in vitro does not affect sFlt-1 measurements using the R&D ELISA kit. Assessment of serum levels with the R&D ELISA after the administration of enoxaparin or vehicle in vitro shows no significant differences in sFlt-1 levels (A and B). There is also no significant difference seen in urinary sFlt1 levels after administration of enoxaparin or vehicle in vitro (C and D). (TIF) [file pone.0085258.s005.tif]
